# Bay 61-3606 Sensitizes TRAIL-Induced Apoptosis by Downregulating Mcl-1 in Breast Cancer Cells

**DOI:** 10.1371/journal.pone.0146073

**Published:** 2015-12-31

**Authors:** So-Young Kim, Sang Eun Park, Sang-Mi Shim, Sojung Park, Kyung Kon Kim, Seong-Yun Jeong, Eun Kyung Choi, Jung Jin Hwang, Dong-Hoon Jin, Christopher Doosoon Chung, Inki Kim

**Affiliations:** 1 ASAN Institute for Life Sciences, ASAN Medical Center, Seoul, Republic of Korea; 2 Department of Biomedical Sciences, Seoul National University, Seoul, Republic of Korea; 3 Department of Radiation Oncology, ASAN Medical Center, University of Ulsan College of Medicine, Seoul, Republic of Korea; 4 Department of Convergence Medicine, University of Ulsan College of Medicine, Seoul, Republic of Korea; 5 Institute for Innovative Cancer Research, ASAN Medical Center, Seoul, Republic of Korea; Sungkyunkwan University, REPUBLIC OF KOREA

## Abstract

Breast cancer cells generally develop resistance to TNF-Related Apoptosis-Inducing Ligand (TRAIL) and, therefore, assistance from sensitizers is required. In our study, we have demonstrated that Spleen tyrosine kinase (Syk) inhibitor Bay 61–3606 was identified as a TRAIL sensitizer. Amplification of TRAIL-induced apoptosis by Bay 61–3606 was accompanied by the strong activation of Bak, caspases, and DNA fragmentation. In mechanism of action, Bay 61–3606 sensitized cells to TRAIL *via* two mechanisms regulating myeloid cell leukemia sequence-1 (Mcl-1). First, Bay 61–3606 triggered ubiquitin-dependent degradation of Mcl-1 by regulating Mcl-1 phosphorylation. Second, Bay 61–3606 downregulates Mcl-1 expression at the transcription level. In this context, Bay 61–3606 acted as an inhibitor of Cyclin-Dependent Kinase (CDK) 9 rather than Syk. In summary, Bay 61–3606 downregulates Mcl-1 expression in breast cancer cells and sensitizes cancer cells to TRAIL-mediated apoptosis.

## Introduction

TRAIL/Apo2 ligand selectively kills cancer cells by initiating apoptotic signaling through the engagement of its pro-apoptotic receptors, Death Receptors-4 and -5 [1, 2]. TRAIL binding to these receptors results in the formation of ‘death-inducing signaling complex (DISC)’ inducing caspase-8 activation [3]. After DISC-mediated activation of caspase-8, the intrinsic apoptotic pathway is activated by processing of Bid and its translocation to mitochondria. The joint effort of the extrinsic and intrinsic apoptotic pathways then leads to the activation of downstream caspases (-3 and -7) and apoptotic demise of cells [4].

Although TRAIL shows cancer-selective killing activity, a phase 2 clinical trial failed to demonstrate a clear benefit in a therapeutic window [5]. Parallel to this result, primary tumors were found to be resistant against TRAIL-induced apoptosis. Resistance to TRAIL is partially explained by decoy receptors (DcR1 and DcR2), which have a deleted or truncated death domain [6]. Other defects of cell death pathways, such as dysregulated expression of anti-apoptotic proteins and pro-apoptotic proteins, were identified as mechanisms of resistance [4, 7]. However, new biomarkers and molecular targets of TRAIL resistance are still needed for its potential future clinical use.

Myeloid cell leukemia sequence-1 (Mcl-1) is a member of the anti-apoptotic Bcl-2 family proteins that neutralizes pro-apoptotic Bcl-2 proteins such as Bim, Bid, and Bad [8]. The important role of Mcl-1 in TRAIL-mediated cell death has been suggested in a number of published studies. Knockdown of the Mcl-1 gene enhances the apoptotic events induced by TRAIL [9, 10]. A recent study of several TRAIL sensitizers revealed that they function *via* downregulation of Mcl-1 [11–14].

Cyclin-Dependent Kinases (CDKs) are a group of protein serine/threonine kinases which is activated by specific cyclin co-factors. Multiple CDKs regulate the cell cycle progression and control the cell death [15]. In fact, several CDK inhibitors, i.e. R-roscovitine, CR8, flavopiridol, and CDKI-73 induce Mcl-1 downregulation and thus promote the induction of apoptosis [16–19]. However, the study that molecular mechanisms and practical approaches downregulate Mcl-1efficiently and safely must still be further clarified.

In this study, we have identified Bay 61–3606 as a new TRAIL sensitizer in MCF-7 breast carcinoma cells. Bay 61–3606 induced ubiquitin (Ub)-dependent degradation of Mcl-1 protein and suppressed mRNA transcription of Mcl-1 by inhibiting Cyclin-Dependent Kinase (CDK9). This result underscores the importance of CDK9-dependent signaling in Mcl-1 downregulation and suggests a new therapeutic strategy to overcome resistance to anti-cancer therapeutics driven by Mcl-1 overexpression.

## Materials and Methods

### Reagents

Recombinant human TRAIL and Lipofectamine 2000 were purchased from Life Technologies (Carlsbad, CA, USA). The CellTiter-Glo viability assay solution was purchased from Promega (Madison, WI, USA). Bay 61–3606, curcumin, and piceatannol were purchased from Sigma Aldrich (St. Louis, MO, USA). Syk inhibitor II and MG-132 were purchased from Calbiochem (San Diego, CA, USA). The following antibodies were used: anti-Bad (CS-9292), anti-Bak (CS-6947), anti-Bax (CS-2772), anti-Bcl-xL (CS-2764), anti-Bid (CS-2002), anti-caspase-7 (CS-9494), anti-caspase-8 (CS-9746), anti-phospho-CDK9 (CS-2549), anti-CDK9 (CS-2316), anti-DR5 (CS-8074), anti-phospho-ERK (CS-4370), anti-ERK (CS-4695), anti-FLIP (CS-8510), anti-phospho-GSK3α/β (CS-9331), anti-GSK3α/β (CS-5676), anti-HA (CS-3724), anti-cIAP1 (CS-7065), anti-Mcl-1 (CS-5453), anti-poly-ADP-ribosyl polymerase (PARP) (CS-9532), anti-RNA polymerase II (CS-2629), anti-phospho-Syk (CS-2711), anti-Survivin (CS-2808), anti-XIAP (CS-2045), and anti-p53 (CS-2527) (Cell Signaling, Danvers, MA, USA). Anti-active Bak (NT 06–536) and anti-Syk (06–486) were purchased from Millipore (Billerica, MA, USA). Anti-phospho-Mcl-1 (AB-111574) and anti-phospho-RNA polymerase II (AB-70324) were purchased from Abcam (Cambridge, MA, USA). Anti-DR4 (SC-7863) was purchased from Santa Cruz Biotechnology (Santa Cruz, CA, USA). Anti-Flag (F1804) and anti-α-Tubulin (T9026) was purchased from Sigma Aldrich (St. Louis, MO, USA).

### Cell Culture

The human breast cancer cells (MCF-7, MDA-MB-231, and T47D) and human kidney cells (293T) were from the ATCC (Manassas, VA, USA). Cells, except for MDA-MB-231, were maintained in RPMI-1640 supplemented with 10% fetal bovine serum (Life Technologies, Carlsbad, CA, USA), _L_-glutamine and 100 U/ml of antibiotic penicillin/streptomycin (Life Technologies, Carlsbad, CA, USA). MDA-MB-231 cells were maintained in DMEM with the same supplements.

### Compound Screening and DNA Fragmentation

High throughput, TRAIL-sensitizer screening and DNA fragmentation assay were performed as described previously [12].

### Western Blot Analysis

Cell extracts were prepared by scraping cells with lysis buffer containing complete protease inhibitor cocktail (Roche, Basel, Switzerland). After protein concentration measurement using the Bio-Rad DC protein assay (Bio-Rad, Veenendaal, Netherlands), extracts were diluted with 2X Laemmli sample buffer. Cell extract (30 μg) was resolved by sodium dodecyl sulfate-polyacrylamide gel electrophoresis (SDS-PAGE) followed by Western blotting using polyvinylidene difluoride membranes (Millipore, Billerica, MA, USA). The membranes were incubated in TRIS-buffered saline with specific antibodies (1:1000 dilutions). The antibody-protein complex was detected with horseradish peroxidase-conjugated IgG (Bio-Rad) and a chemiluminescent substrate solution (SuperSignal West Pico; Pierce, Rockford, IL). Stripped membranes were then re-probed with antibody to α-tubulin as a loading control.

### Cell Viability and Immunocytochemistry

For the quantitative assay of cell survival, a cellular, ATP-based, luminescent kit (CellTiter-Glo; Promega, Madison, WI, USA) was used according to the manufacturer’s protocol. The raw luminescence value was measured and the relative cell survival was calculated using the GraphPad Prism software (La Jolla, CA, USA). For immunocytochemistry, an antibody raised against active Bak was used to detect apoptosis. Briefly, cells were seeded on a chamber slide. After treatment, cells were fixed with 4% paraformaldehyde, then permeabilized with 0.5% Triton X-100 in PBS, and blocked with 10% goat serum diluted in PBS. The slide was incubated with primary antibody and secondary antibody with washing steps. After mounting, the sample image was visualized under fluorescence microscopy (Olympus LX71; Tokyo, Japan).

### Expression Constructs, Transient Transfection, and Luciferase Activity Assays

pBabe-flag-Mcl-1 [20], pcDNA3-CDK9-HA, pcDNA3-CDK9 (DN)-HA, pGL2-basic, and pGL2-Mcl-1-luc promoter [11] plasmids were acquired from Addgene (Cambridge, MA, USA). One day before transfection, cells were plated at a density of 5 × 10^5^ cells in 6-well plates. Cells were transfected with expression plasmids using Lipofectamine 2000 (Invitrogen, Carlsbad, CA, USA). For luciferase assays, MCF-7 cells were co-transfected with plasmids containing luciferase reporter genes (pGL2-Mcl-1-luc and pRL-TK-luc) using Lipofectamine 2000 for 48 h. Cells were pre-incubated with Bay 61–3606 (2.5 μM) for 1 h and were then exposed to TRAIL (50 ng/ml) for an additional 6 h. After treatment, cells were lysed with lysis buffer and aliquots of the lysates were used to analyze the transcription level of Mcl-1 using the Dual-Luciferase Reporter Assay kit (Promega, Madison, WI, USA).

### Caspase-7 and -8 Activity Assay

The activity of caspase-7 and -8 were measured using a fluorometric activity assay system (R&D system, MA, USA). After 1 h of pre-incubation with Bay 61–3606 (5 μM), cells were exposed to TRAIL (50 ng/ml) for 24 h. Cells were harvested using lysis buffer, after which cell lysates were incubated with caspase-7 (DEVD-AFC) or caspase-8 (IETD-AFC) substrate for 1 h at 37°C. The fluorescent signal was measured using Wallac Victor 3 label reader (Perkin Elmer, Waltham, MA, USA).

### siRNA Transfection

Mcl-1 siRNA (5’-AAGUAUCACAGACGUUCUCTT-3’), Syk siRNA (5’-CGCUCUUAAAGAUGAGUUATT-3’), and a control siRNA (5’-ACGUGACACGUUCGGAGAAUU-3’) were designed and synthesized by Genolution Corp (Seoul, Korea). CDK9 siRNA #1 (5’-GGGAGAUCAAGAUCCUUCAGCUUCU-3’), and CDK9 siRNA #2 (5’-UGGUCAAGUUCACGCUGUCUGAGAU-3’) were purchased from Life Technologies (Carlsbad, CA, USA). CDK9 siRNA #3 (5’-GACUUGCAUCGUGGAGACA-3’) were designed and synthesized by Bioneer (Daejeon, Korea). Cells were transfected with RNAiMAX^™^ (Life Technologies, Carlsbad, CA, USA) according to the manufacturer’s protocol. The cell viability was examined using trypan blue exclusion, and knockdown efficiency was confirmed by western blotting.

### Reverse Transcription Polymerase Chain Reaction (RT-PCR)

Total RNA of test cell was isolated by the TRI reagent (Molecular Research Center, Cincinnati, OH, USA), and the cDNA was prepared using RevertAid First Strand cDNA synthesis kit (Life Technologies, Carlsbad, CA, USA). PCR was performed with specific primers: Mcl-1 (sense) 5’-TAAGGACAAAACGGGACTGG-3’ and (antisense) 5’-ACCAGCTCCTACTCCAGCAA-3’; SYK (sense) 5’-GTGTTGCTAGTTACCCAACATTACGC-3’ and (antisense) 5’-AGTTCACCACGTCATAGTAGTAATT-3’;, and GAPDH (sense) 5’-GAGTCAACGGATTTGGTCGT-3’ and (antisense) 5’-TTGATTTTGGAGGGATCTCG-3’. The PCR protocol is as followed: 95°C for 5 min, followed by 25 cycles of 95°C for 1 min, 60°C for 1 min, 72°C for 1 min, and a final extension at 72°C for 7 min. The relative expression of Mcl-1 mRNA was normalized *versus* GAPDH.

### Ubiquitination

293T cells were transfected with plasmids encoding Flag-Mcl-1 and HA-Ub using Lipofectamine 2000 for 48 h. Cells were incubated with MG-132 or Bay 61–3606 for 6 h, lysed with lysis buffer containing 0.5 μM iodoacetate, 20 mM N-ethylmaleimide (NEM), and 5 μM MG-132. After centrifugation, Flag-tagged Mcl-1 was immunoprecipitated by anti-Flag antibody and protein-A agarose beads. Ubiquitination of Flag-Mcl-1 protein was visualized by Western blotting using anti-HA and anti-Flag antibody.

### *In Vivo* Xenograft Model Assay

Female BALB/c nude mice (5 weeks old) were used (Japan SLC Inc., Shizuoka, Japan) to generate a tumor xenograft model. For each mouse, a β-estradiol 17-valerate pellet (0.5 mg, 60 days release, Innovative Research of America) was subcutaneously implanted into the neck region to support tumor growth following the implantation of 1 × 10^6^ MCF-7 cells subcutaneously into the hind limb. Formation of the tumor and condition of animal were monitored every other day. When the tumor volume reached ~100 mm^3^, the mice were divided into four treatment groups (n = 5 / group), i.e. untreated, TRAIL (10 mg/kg), Bay 61–3606 (50 mg/kg), and a combination of Bay 61–3606 and TRAIL. TRAIL with or without Bay 61–3606 was administered twice a week for two weeks by intraperitoneal injection. TRAIL was given 2 h after the injection of Bay 61–3606. The largest longitudinal (length) and transverse (width) tumor diameters were measured using a vernier caliper, and the tumor volume was calculated with the formula: *tumor volume = 1/2 (length × width* ^*2*^ *)*.

### Ethics Statement

All animal experiments were performed under the guide protocol approved by the Institutional Animal Care and Use Committee of the ASAN Institute for Life Sciences and performed in accordance with the institution guidelines. All efforts were made to minimize suffering. For the implantation procedure, animals were anaesthetized by intraperitoneal injection of 1.2% Tribromoethanol at 125~250 mg/kg and recovered on heating pad. Animals were euthanized by CO_2_ inhalation in a prefilled CO_2_ chamber at the end of designated experiment, or to alleviate untreatable pain or distress such as tumor burden exceeding 10% of body weight, weight loss greater than 20% and other severe clinical signs.

### Statistical Analysis

All the experiments were independently repeated at least two or more times, data were presented as the mean ± SD using a Two-way ANOVA with software by GraphPad Prism 5.0., and *P*-values < 0.05 were considered statistically significant.

## Results

### Bay 61–3606 Sensitizes MCF-7 Cells to TRAIL-Induced Apoptosis

**Fig 1:**
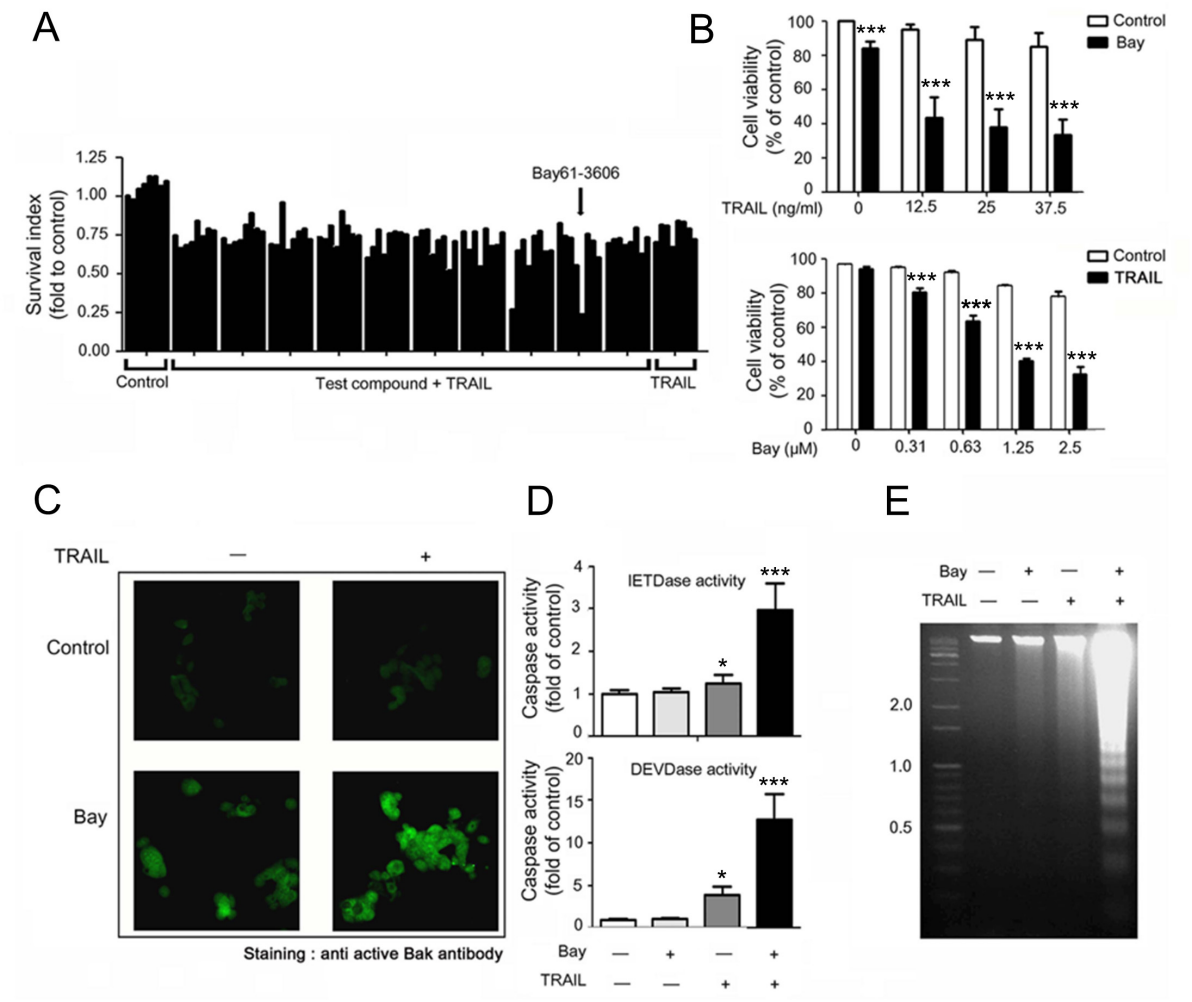
Bay 61–3606 is a sensitizer of TRAIL-induced apoptosis. (A) Assay plate where Bay 61–3606 was identified as a sensitizer of TRAIL. After pre-incubation of test compounds (5 μM), cells were exposed to TRAIL (50 ng/ml). Cell survival was measured as a percentage of the control sample. The arrow indicates Bay 61–3606 (5 μM). (B) MCF-7 cells were exposed to TRAIL (indicated concentrations) with or without Bay 61–3606 (2.5 μM) for 24 h (upper) and Bay 61–3606 (indicated concentrations) with or without TRAIL (50 ng/ml) for 24 h (lower). (C) After exposure to the agents for 12 h, MCF-7 cells were subjected to immunocytochemistry using an active Bak antibody. (D) Caspase activity in MCF-7 cells exposed to Bay 61–3606 (5 μM) with or without TRAIL (50 ng/ml) for 24 h. (E) DNA fragmentation in MCF-7 cells was treated as seen in (D). Values are the mean ± SE from three separate experiments performed in triplicate. Asterisks indicate significant differences compared with the control (* *P* <0.05, and *** *P*-values < 0.001).

To identify new sensitizers of TRAIL, we performed a compound screen using a library of 1,280 functionally characterized small molecules (LOPAC-1280) in MCF-7 cells. Eight candidates listed in S1 Table could sensitize MCF-7 cells to a subtoxic dose of recombinant TRAIL. Of these, Bay 61–3606 sensitized cells to death induced by TRAIL while exposure to TRAIL alone showed minimal reduction (Fig 1A, bars in far right). Combination treatment induced synergistic cell death in a concentration-dependent manner (Fig 1B). To characterize the type of death, cells were subjected to immunocytochemistry analysis (Fig 1C), caspase activity assay (Fig 1D), and DNA fragmentation assay (Fig 1E). Upon combination treatment, Bak and caspases-7 & -8 were activated and DNA was potently fragmented. All of the qualitative cell death assays revealed that Bay 61–3606 sensitized MCF-7 cells to TRAIL-induced cell death with apoptotic characteristics. Moreover, treatment with a variety of cell death-inducing agents, Bay 61–3606 is specific for the death receptor-mediated cell death signaling, i.e. TNF-α (S1A Fig). And Bay 61–3606 sensitized all other cancer cells to TRAIL in a synergistic or additive manner (S1B Fig).

### Bay 61–3606 Sensitizes MCF-7 Cells to TRAIL-Induced Apoptosis by Mcl-1 Down-Regulation

**Fig 2:**
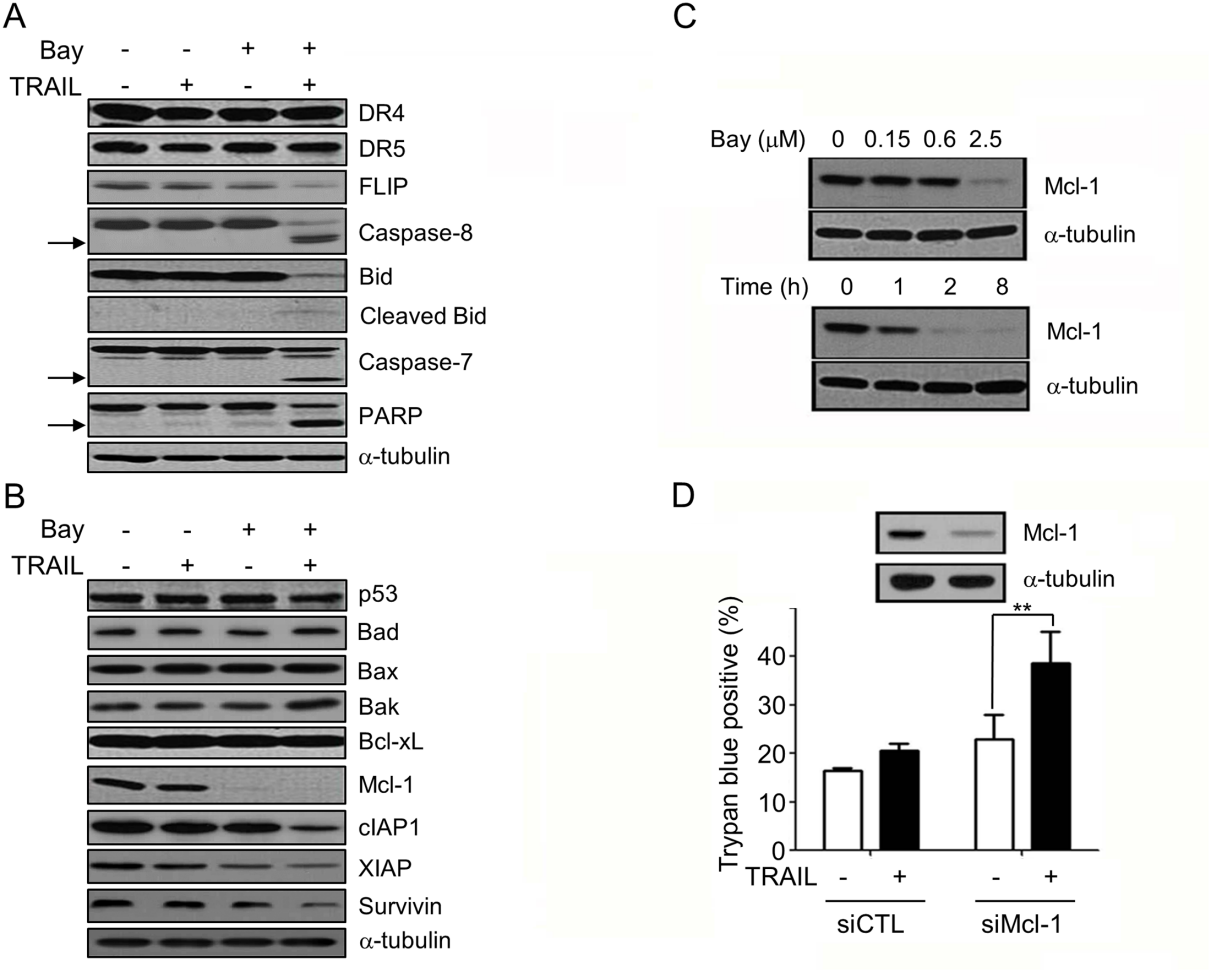
Bay 61–3606 sensitizes MCF-7 cells to TRAIL by decreasing Mcl-1. (A, B) After MCF-7 cells were exposed to TRAIL (50 ng/ml) with or without Bay 61–3606 (2.5 μM) for 12 h, cells were extracted and analyzed by immunoblotting analysis using the indicated antibodies. (C) MCF-7 cells were exposed to several concentrations of Bay 61–3606 (upper) for 6 h or to 2.5 μM Bay 61–3606 for the indicated times (lower), and expression of Mcl-1 was analyzed by Western blotting. (D) MCF-7 cells were transfected with scrambled (siCTL) or Mcl-1 siRNA (siMcl-1). After 48 h, cells were exposed to TRAIL (50 ng/ml) for 24 h. The percentage of cell death was evaluated by trypan blue exclusion. Values are the mean ± SE from three separate experiments performed in triplicate. Asterisks indicate significant differences compared with the control (** *P*-values < 0.01).

We then analyzed the molecular mechanisms of the sensitization. The combination of Bay 61–3606 and TRAIL massively induced caspase-7 & -8 activation and cleavage of Bid, and PARP (Fig 2A). As shown in Fig 2A and 2B, there was no significant alteration in the expression levels of DR4, DR5, p53, and Bcl-2 family proteins, Bad, Bax, Bak, and Bcl-xL in MCF-7 cells. However, we could detect significantly Mcl-1 downregulation by Bay 61–3606 (Fig 2B) and Mcl-1 downregulation were concentration- and time-dependent (Fig 2C). Also, FLICE-inhibitory protein (FLIP) and Inhibitors of Apoptosis Proteins (IAP) family proteins, such as cIAP1, X-linked inhibitor of apoptosis protein (XIAP), and Survivin were downregulated by combination of Bay 61–3606 and TRAIL (Fig 2A and 2B). To examine the contribution of Mcl-1 to TRAIL resistance, the loss of function effect of Mcl-1 was tested. As shown in Fig 2D, Mcl-1 clearly knock downed the sensitized cells to TRAIL, implying that Mcl-1 contributes to the resistance to TRAIL. These results indicate that Bay 61–3606 sensitized MCF-7 cells to TRAIL by decreasing Mcl-1. Mcl-1 clearly knock downed the sensitized cells to TRAIL, implying that Mcl-1 contributes to the resistance to TRAIL. These results indicate that Bay 61–3606 sensitized MCF-7 cells to TRAIL by decreasing Mcl-1.

### Bay 61–3606 Induces Mcl-1 Downregulation in a Syk-Independent Manner

**Fig 3:**
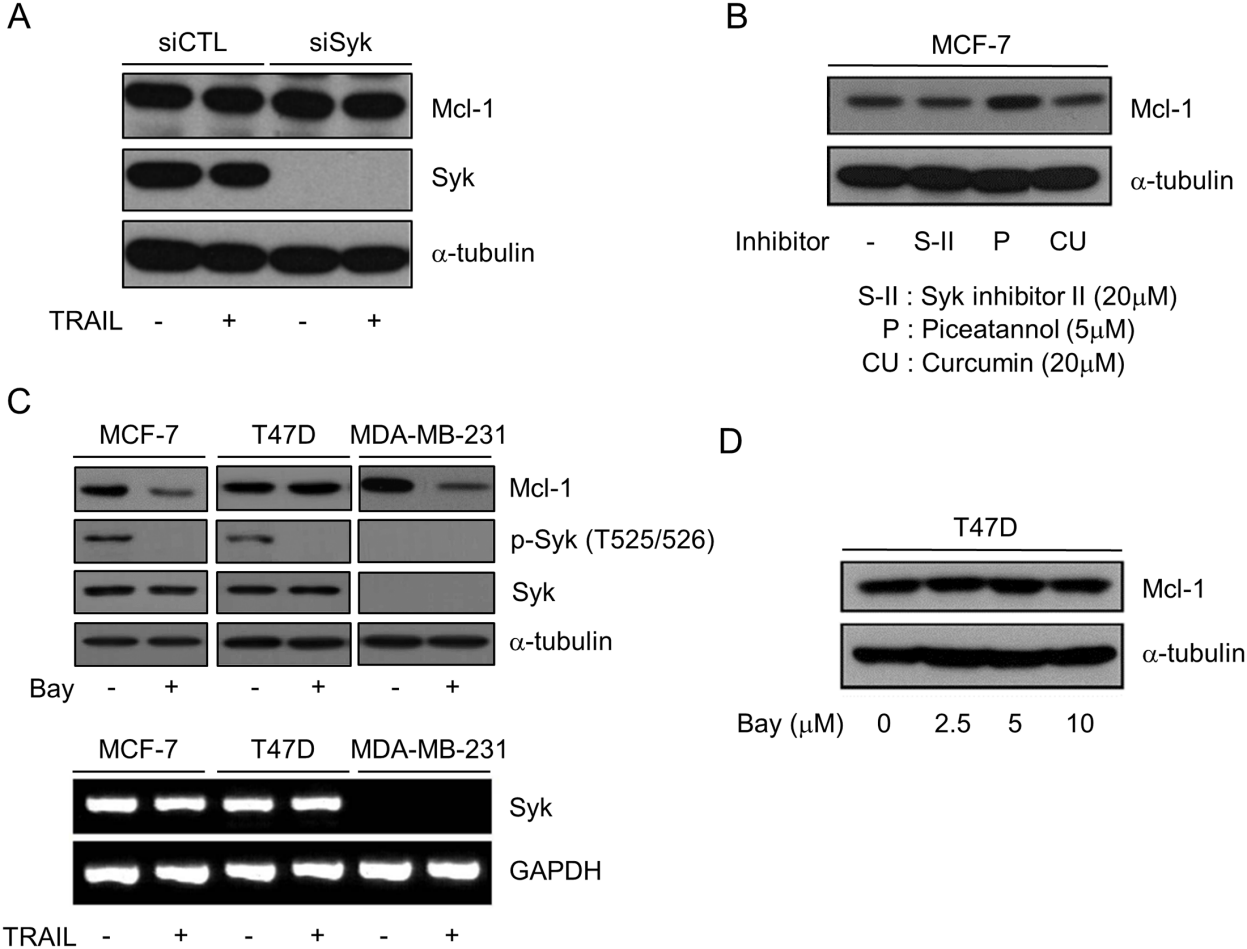
Mcl-1 degradation by Bay 61–3606 is independent of Syk. (A) MCF-7 cells were transfected with scrambled (siCTL) or Syk siRNA (siSyk) for 48 h, and expression of Syk and Mcl-1 was analyzed by Western blotting. (B) MCF-7 cells were exposed to Syk inhibitors for 6 h and Mcl-1 expression was assessed by Western blotting. (C) Three, breast-cancer cell lines were exposed to TRAIL (50 ng/ml) for 6 h, Syk mRNA expression was analyzed by RT-PCR (lower), and phosphorylation of Syk and expression of Mcl-1 were analyzed by Western blotting(upper). (D) T47D cells were exposed to increasing concentrations of Bay 61–3606 for 6 h, and expression of Mcl-1 was analyzed by Western blotting.

Bay 61–3606 is an inhibitor of spleen tyrosine kinase [21, 22], although it has a wide-ranging inhibitory profile against various kinases [23]. These multiple target spectra of Bay 61–3606 led us to investigate whether Syk could be involved in Bay 61-3606-mediated downregulation of Mcl-1. First, we examined whether downregulation of Syk expression can affect the Mcl-1 expression. Although we efficiently suppressed Syk expression *via* siRNA, the protein levels of Mcl-1 remained unchanged in MCF-7 cells (Fig 3A). In addition to genetic alteration using siRNA, we also tested three chemical inhibitors of Syk, i.e. S-II [2-(2-aminoethylamino)-4-(3-trifluoromethylanilino)-pyrimidine-5-carboxamide dihydrochloride dehydrate], Piceatannol, and curcumin [24, 25]. In our test, none of these Syk inhibitors reduced Mcl-1 expression in MCF-7 cells (Fig 3B).

The association of Syk with Mcl-1 expression was also tested in other breast cancer cells with different genetic backgrounds. The mRNA and protein level of Syk were detected in both of MCF-7 and T47D cells, and phosphorylation of Syk was reduced by Bay 61–3606 in both cells. But MDA-MB-231 cell was Syk negative in mRNA and protein expression (Fig 3C). As shown in Fig 3C, we re-noted that Mcl-1 protein was reduced in MCF-7 cells by Bay 61–3606, however Bay 61–3606 failed to reduce Mcl-1 in Syk-positive T47D cells though Bay 61–3606 was successful to inhibit Syk phosphorylation. Even in high concentration, Bay 61–3606 failed to reduce Mcl-1 protein in T47D cells (Fig 3D). In contrast, Bay 61–3606 attenuated Mcl-1 expression in MDA-MB-231 cell where Syk expression is not detected (Fig 3C). In conclusion, these data suggest that downregulation of Mcl-1 by Bay 61–3606 is independent of Syk in breast cancer cells.

### Bay 61–3606 Promotes the Ubiquitin/Proteasome-Dependent Degradation of the Mcl-1 Protein in MCF-7 Cells

**Fig 4:**
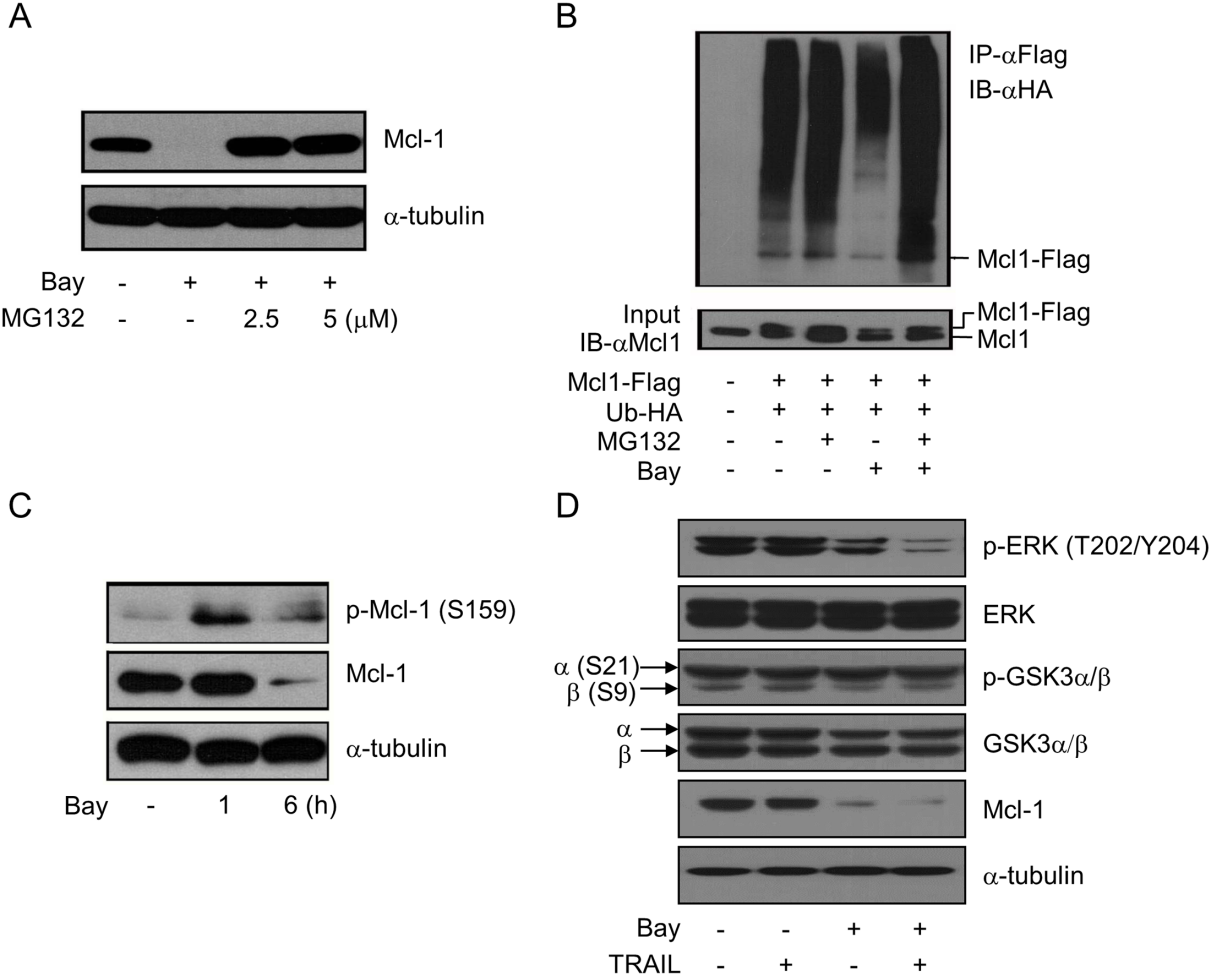
Bay 61–3606 induced ubiquitin-dependent degradation of Mcl-1 by ERK inactivation. (A) MCF-7 cells were exposed to MG132 for 1 h and incubated with 2.5 μM Bay 61–3606 for 6 h. Mcl-1 expression was analyzed by Western blotting. (B) MCF-7 cells were transfected with pcDNA-Mcl1-flag (Mcl1-Flag) vector with pcDNA-Ubiquitin-HA (Ub-HA) vector. After 48 h of transfection, Ubiquitin-conjugated Mcl-1 was immunoprecipitated with anti-Flag antibody, and then detected using anti-HA antibody (upper). Transient expression of exogenous Mcl-1 was confirmed by Western blotting using the Mcl-1 antibody (lower). (C, D) MCF-7 cells were exposed to Bay 61–3606 (2.5 μM) alone (C) or in combination with TRAIL (50 ng/ml) for 6 h (D). The expressions and phosphorylations of Mcl-1 (C) or ERK, and GSK3α/β (D) were determined by Western blotting.

As Mcl-1 is readily degraded by the proteasome [26, 27], we investigated whether Bay 61-3606-mediated Mcl-1 loss could be inhibited by MG-132, an inhibitor of the 26S proteasome. Pre-incubation of cells with MG-132 prevented the loss of Mcl-1 by Bay 61–3606 (Fig 4A). To support the premise that Bay 61–3606 triggers ubiquitination of Mcl-1, we investigated whether Bay 61–3606 could increase the ubiquitination of Mcl-1 protein (Fig 4B). As shown in Fig 4B, pre-incubation with Bay 61–3606 dramatically reduced both the amount of Mcl-1 and that of ubiquitinated Mcl-1 (fourth lane), while pre-incubation with MG132 before adding Bay 61–3606 led to the accumulation of both Mcl-1 and ubiquitinated Mcl-1 (fifth lane). Taken together, this result indicates that Bay 61–3606 triggers the degradation of Mcl-1 by enhancing the ubiquitin-proteasome system (UPS). Phosphorylation of Ser159 in Mcl-1 was mediated by the AKT/GSK3 pathway and therefore increased ubiquitination and degradation of Mcl-1 [28]. Therefore, we assessed the phosphorylation status of Mcl-1 after exposure to Bay 61–3606. We detected a rapid increase of Mcl-1 phosphorylation at Ser159 after 1 h of Bay 61–3606 incubation and then increased Mcl-1 degradation after exposure to Bay 61–3606 for 6 h (Fig 4C). However, GSK3 phosphorylation was not modulated by Bay 61–3606 (Fig 4D). These results show that Bay 61–3606 induces the degradation of Mcl-1 by phosphorylation at Ser159 independently from GSK3.

In addition, phosphorylation of Thr163 in the Mcl-1 PEST domain, which is an Mcl-1 stabilizing event, is mediated by ERK [29–31]. As shown in Fig 4D, treatment with Bay 61–3606 reduced the phosphorylation of ERK, implying that Bay 61–3606 inhibits ERK activity. Moreover, the combination of Bay 61–3606 and TRAIL enhanced ERK inactivation and degradation of Mcl-1. To a better understanding of the biochemical changes in Mcl-1 related to its degradation, time-dependent changes in phosphorylation of Ser159 and Thr163 residues were also analyzed (S2 Fig). Within 1 h after incubation, phosphorylations at both of Ser159 and Thr163 residues reached the maximum level followed by a rapid decrease. At this point, the phosphorylation enhancing patterns were similar to each other. Taken together, our data suggest that Bay 61–3606 enhances degradation of Mcl-1 *via* increased phosphorylation at Ser159 and the UPS and by inhibiting ERK phosphorylation.

### Bay 61–3606 Inhibits the Phosphorylation of CDK9, RNA Polymerase II, and Mcl-1 Expression in MCF-7 Cells

**Fig 5:**
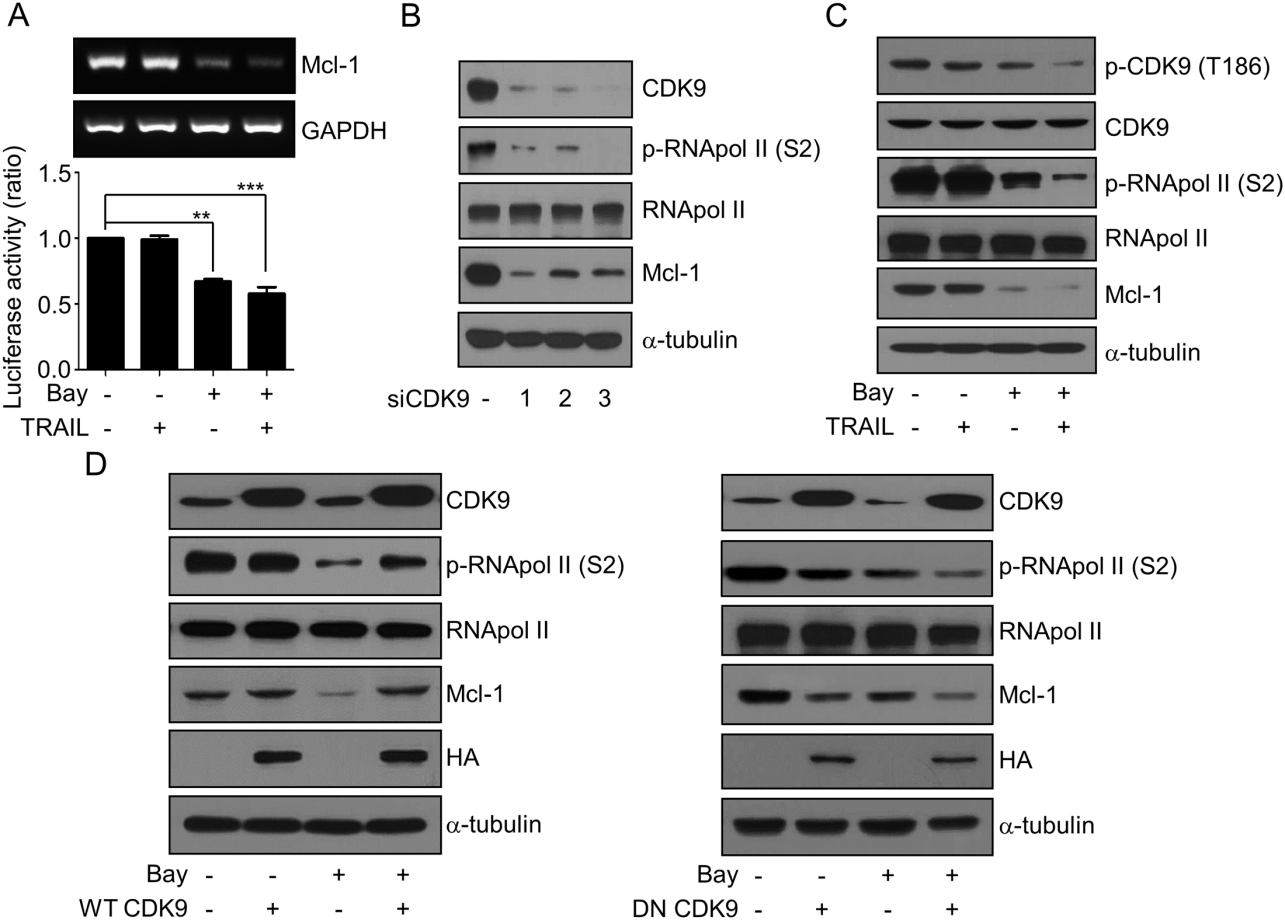
Bay 61–3606 downregulated Mcl-1 expression by inhibition of CDK9 and RNA polymerase II phosphorylation. (A) Cells were treated by Bay 61–3606 (2.5 μM) combined with TRAIL (50 ng/ml) for 6 h, and transcriptional downregulation of Mcl-1 was measured by RT-PCR (A, upper), and Mcl-1 luciferase reporter assay (A, lower). (B) MCF-7 cells were transfected with scrambled siRNA or three different CDK9 siRNAs (siCDK9 #1, #2, and #3). After 48 h of transfection, the expressions of CDK9, RNA pol II, Mcl-1 with phosphorylation of RNA pol II were analyzed by Western blotting. (C) Cell extracts were prepared from cells treated with in (A) and the expressions of CDK9, RNA pol II, Mcl-1 with phosphorylation of RNA pol II were analyzed by Western blotting. (D) MCF-7 cells were transfected with pcDNA3-WT-CDK9-HA or pcDNA3-DN-CDK9-HA. After 48 h of transfection, cells were treated with Bay 61–3606 (2.5 μM) for 6 h. Cell extracts were then analyzed by Western blotting using the indicated antibodies. Values are the mean ± SE from three separate experiments performed in triplicate. Asterisks indicate significant differences compared with the control (** *P*-values < 0.01, and *** *P*-values < 0.001).

We investigated whether Bay 61–3606 modulates Mcl-1 expression at the transcription level. As shown in Fig 5A, downregulation of Mcl-1 mRNA expression by Bay 61–3606 was examined by RT-PCR and the Mcl-1 promoter assay. Moreover, Bay 61–3606 inhibited CDK9 kinase activity with an *in vitro* IC_50_ of 37 nM (S3 Fig). Based on this result, we investigated the molecular mechanisms in which Bay 61–3606 inhibits Mcl-1 transcription *via* CDK9. As shown in Fig 5B, knockdown of CDK9 by siRNAs inhibited RNA polymerase II (RNA pol II) phosphorylation at Ser2 and induces Mcl-1 downregulation. Incubation with Bay 61–3606 promoted the inhibition of CDK9 and RNA pol II phosphorylation, and resulted in Mcl-1 downregulation regardless of the TRAIL treatment (Fig 5C). This result is comparable with knockdown of CDK9 induces Mcl-1 downregulation by RNA pol II inhibition (Fig 5B), and thus implying that Bay 61–3606 has a negative role on CDK9 and RNA pol II activity.

Moreover, two CDK9 specific inhibitors significantly inhibited simultaneous phosphorylation of CDK9 and RNA pol II and they induced Mcl-1 downregulation in a dose-dependent manner as Bay 61–3606 (S4 Fig). We found that downregulation of Mcl-1 by Bay 61–3606 was reversed by wild type CDK9 (WT CDK9) overexpression (Fig 5D left). Also WT CDK9 reversed the decreased phosphorylation of RNA pol II by Bay 61–3606. In contrast to our WT CDK9 and the kinase-inactive, CDK9 dominant-negative mutant (DN CDK9) decreased RNA pol II phosphorylation and reduced Mcl-1 protein even without Bay 61–3606 treatment. In addition, overexpression of DN CDK9 with Bay 61–3606 incubation together reduced Mcl-1 protein expression and enhanced RNA pol II dephosphorylation by Bay 61–3606 (Fig 5D right). These results suggest that Bay 61–3606 reduces Mcl-1gene transcription by inhibiting CDK9-dependent RNA pol II activation.

### Anti-Tumor Activity of TRAIL with Bay 61–3606 *In Vivo*

**Fig 6:**
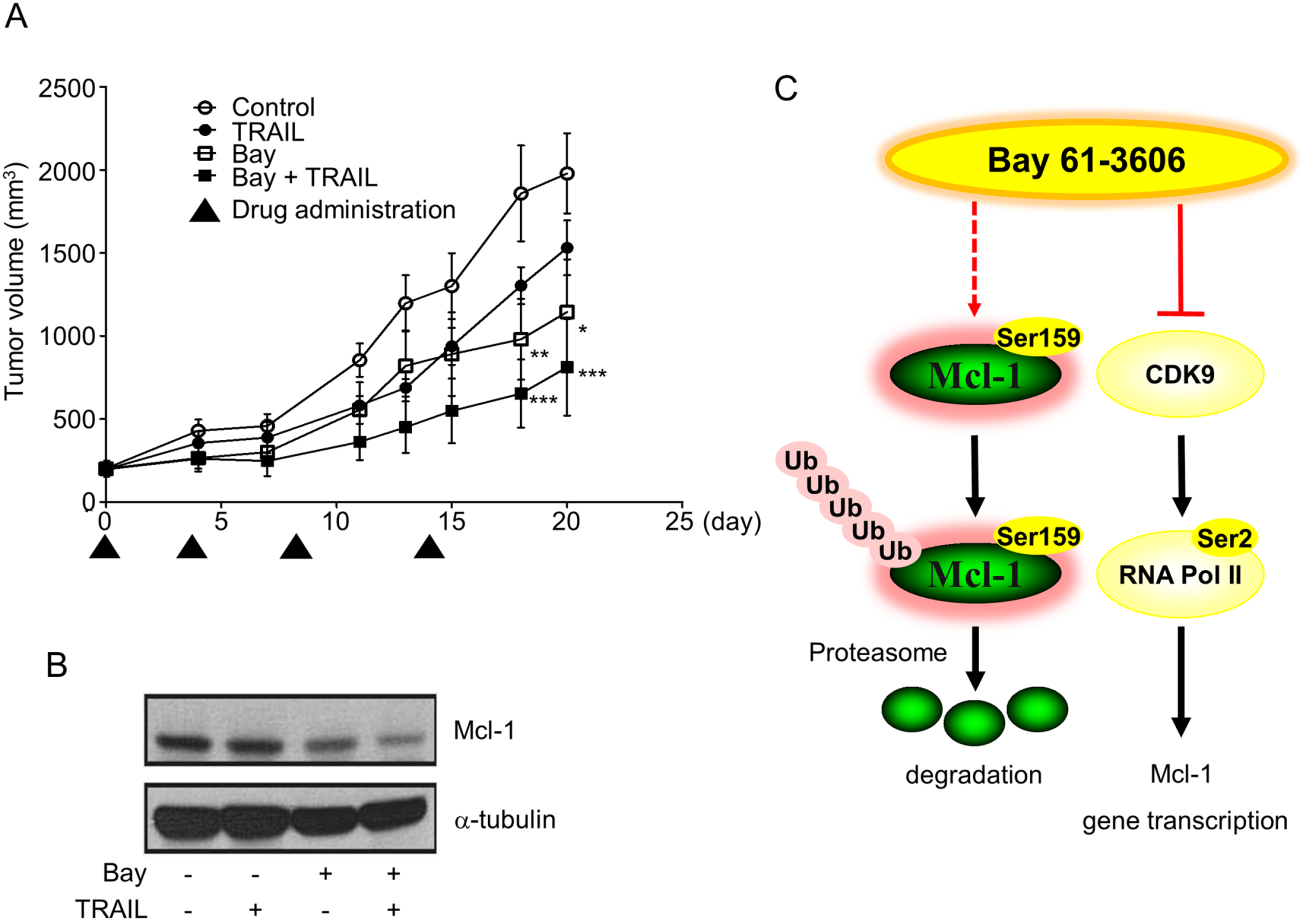
The anti-tumor effect of Bay 61–3606 and TRAIL *in vivo*. (A) The mean tumor volumes are presented. After the average volumes of the tumors reached ~100 mm^3^, mice (n = 5/group) were injected intraperitoneally twice a week with TRAIL (10 mg/kg), Bay 61–3606 (50 mg/kg) or a combination of Bay 61–3606 (50 mg/kg) and TRAIL (10 mg/kg). Asterisks indicate significant differences compared with the control (* *P* <0.05, ** *P* <0.01, and *** *P* <0.001). (B) Protein extracts from tumor xenografts were analyzed for Mcl-1 expression by Western blotting after mice were sacrificed. (C) A suggested model for Bay 61–3606 for sensitizing TRAIL-induced apoptosis by downregulating Mcl-1 in breast cancer cells.

The *in vivo* anti-tumor activity of Bay 61–3606 was assessed by treating MCF-7 tumor xenograft-bearing BALB/c nude mice with a combination of Bay 61–3606 and TRAIL (Fig 6A). Interestingly, the TRAIL single agent led to slight reductions in tumor growth and the administration of Bay 61–3606 alone (*P* <0.05) was more efficacious than that of TRAIL alone. After 20 days of drug administration, the volume of the xenografted tumor was significantly (*P* <0.001) reduced the efficacy of Bay 61–3606 when administered in TRAIL combination. Bay 61–3606 reduced Mcl-1 protein using tissue extract by Western blot analysis (Fig 6B). These results suggest that combination treatment with Bay 61–3606 and TRAIL suppresses tumor growth *in vivo*.

## Discussion

This study examined whether Bay 61–3606 sensitizes cells to TRAIL-induced apoptosis and if so, clarified the detailed molecular mechanisms in human breast cancer cells. Our results provide a hypothetical mechanism regarding how Bay 61–3606 downregulates the Mcl-1 protein level and gene transcription (Fig 6C). First, we have shown that Bay 61–3606 induces Mcl-1 protein degradation by phosphorylating Ser159. The phosphorylated Mcl-1 at Ser159 interacts with ubiquitin-ligase and promotes proteasomal degradation [32–34]. We could also detect inactivation of ERK by Bay 61–3606, and thus implying that Mcl-1 was freed from ERK-mediated stabilization signaling. However, Mcl-1 phosphorylation at Ser159 by Bay 61–3606 does not depend on the GSK3 differently from that shown in previously published reports showing that ERK and GSK are important regulators in Mcl-1 stability (Fig 4) [28, 31, 32]. This suggests that other upstream kinases regulate Mcl-1 stabilization and degradation by multiple alternative routes.

Secondly, we found that Bay 61–3606 inhibits Mcl-1 gene transcription by inhibiting CDK9-mediated RNA pol II activity. To address the effect of Bay 61–3606 on Mcl-1, we suspected that Syk plays essential role to regulate Mcl-1 in cells because Bay 61–3606 was originally developed for the inhibitor of Syk [21]. However, we have determined that Syk is not involved in the Bay 61-3606-mediated Mcl-1 downregulation (Fig 3). This result is comparable with that of previous reports indicating that Syk is a tumor suppressor in breast cancer cells [35]. Meanwhile, we noticed that Bay 61–3606 interacts with CDK9 at the ATP binding site [23] and inhibits CDK9 kinase activity. In our data, we could show that CDK9 inhibition results in RNA pol II dephosphorylation at Ser2 and the downregulation of Mcl-1. The combination of Bay 61–3606 and TRAIL significantly decreased the phosphorylation of CDK9 and RNA pol II. Moreover, knockdown of CDK9, specific CDK9 inhibitors, and kinase inactivation of CDK9 (Fig 5B–5D and S4 Fig) can strongly suppress RNA pol II-mediated Mcl-1 transcription and can decrease Mcl-1 expression similarly to that of Bay 61–3606. In addition, the direct inhibition of CDK9 with RNA pol II inactivation by Bay 61–3606 has been suggested (Fig 5C) and CDK9 is the main target of Bay 61–3606 to control Mcl-1. Our results are consistent with those of previous reports regarding roscovitine [17] and flavopiridol, both of which are inhibitors of CDK9 [18]. It has recently been shown that a novel CDK9 inhibitor inhibits RNA pol II phosphorylation and Mcl-1 transcription, thus showing strong anti-cancer therapeutic activity [19]. All this taken together, CDK9 is an important target kinase that control Mcl-1 stability and turnover in cancer cells. Future studies regarding the detailed mechanisms of Mcl-1 regulation *via* CDK9 may help to develop a new targeted therapeutic strategy for cancer.

Although these two events suggest Bay 61-3606-mediated Mcl-1 degradation regulation, there still are unanswered questions regarding the detail mechanism of Mcl-1 degradation by Bay 61–3606. Most of all, GSK3 phosphorylation was not modulated by Bay 61–3606, in contrast to ERK. Because GSK3 was reported as the main regulator of Mcl-1 Ser159 phosphorylation, it should be clarified how Bay 61–3606 can induce Ser159 phosphorylation without special activation of GSK3. The second issue regarding Mcl-1 degradation by Bay 61–3606 is the balancing between Ser159 and Thr163 phosphorylation. As S2 Fig shows, Bay 61–3606 induces both of Ser159 and Thr163 phosphorylation in a similar, time-dependent manner. Because each of these two phosphorylation events represents opposite results for Mcl-1 (degradation *vs* stabilization), it can be expected that there is a more delicate mechanism to regulate the balance between stable and fragile Mcl-1 over simple phosphorylation induction. Lastly, as Fig 2B shows, the anti-apoptotic proteins including Flip and IAP family proteins were similarly downregulated by Bay 61–3606 requesting more detail mechanistic investigation.

In conclusion, we suggest that Bay 61–3606 is a promising agent for downregulating Mcl-1 expression in cancer cells. Our results also strongly suggest that Bay 61–3606 reduces Mcl-1 mRNA transcription by inhibition of CDK9-dependent RNA pol II activation and induces rapid protein degradation *via* the UPS. Based on these results, specific CDK9 inhibitors and identification of a new Bay 61–3606 target for Mcl-1 degradation may be beneficial for the treatment of cancers in which Mcl-1 contributes to the development of resistance to anticancer therapeutics.

## Supporting Information

S1 Fig Specificity of sensitization by Bay 61–3606. (A) Cells were exposed to Bay 61–3606 (2.5 μM) for 1 h in a 96-well plate, after which they were exposed to various cell-death-inducing agents including 125 nM staurosporine (STS), 5 μM daunorubicin (DA), 800 nM etoposide (ETO), 10 μM thapsigargin (TG), 625 ng/ml tunicamycin (TUN), 2.5 ng/ml paclitaxel (PAC), 312.5 nM 2-deoxyglucose (2-DG) or 40 ng/ml tumor necrosis factor alpha (TNF-α) for 24 h. (B) The Bay 61-3606-induced sensitization was tested in SK-BR-3, MDA-MB-231, and AsPC-1 cells which are resistant to TRAIL (50 ng/ml). Asterisks indicate significant differences compared with the control (* *P* <0.05, ** *P* <0.01, and *** P <0.001).
(TIF)

S2 Fig Bay 61–3606 downregulates Mcl-1 by regulating ERK, RNA polymerase II, and Mcl-1 phosphorylation. MCF-7 cells were exposed to Bay 61–3606 (2.5 μM) for increasing times, and cell extracts were analyzed by Western blotting using the indicated antibodies.
(TIF)

S3 Fig CDK9 kinase inhibition of Bay 61–3606. Kinase activity and *in vitro* IC_50_ were determined by Merck Millipore’s Kinase Profiling Service. Protein kinase was tested in a radiometric assay format, and the raw data was measured by scintillation counting.
(TIF)

S4 Fig Inhibition of CDK9 reduces Mcl-1 expression by inhibition of CDK9 and RNA polymerase II phosphorylation. MCF-7 cells were treated with the indicated concentrations of two CDK9 inhibitors (I and II) for 1 h, after which cell extracts were analyzed by Western blotting using the indicated antibodies.
(TIF)

S1 Table Candidate compounds reproducibly sensitizing MCF-7 cells to TRAIL. After pre-incubation of the test compounds (5 μM), cells were exposed to TRAIL (50 ng/ml) for 24 h. Relative cell survival was determined by assaying the ATP levels.
(TIF)
