# Supplementary figures and images for: Bay 61-3606 Sensitizes TRAIL-Induced Apoptosis by Downregulating Mcl-1 in Breast Cancer Cells
Source: PLoS One. 2015 Dec 31;10(12):e0146073. doi: 10.1371/journal.pone.0146073 (PMC4697837; doi:10.1371/journal.pone.0146073)

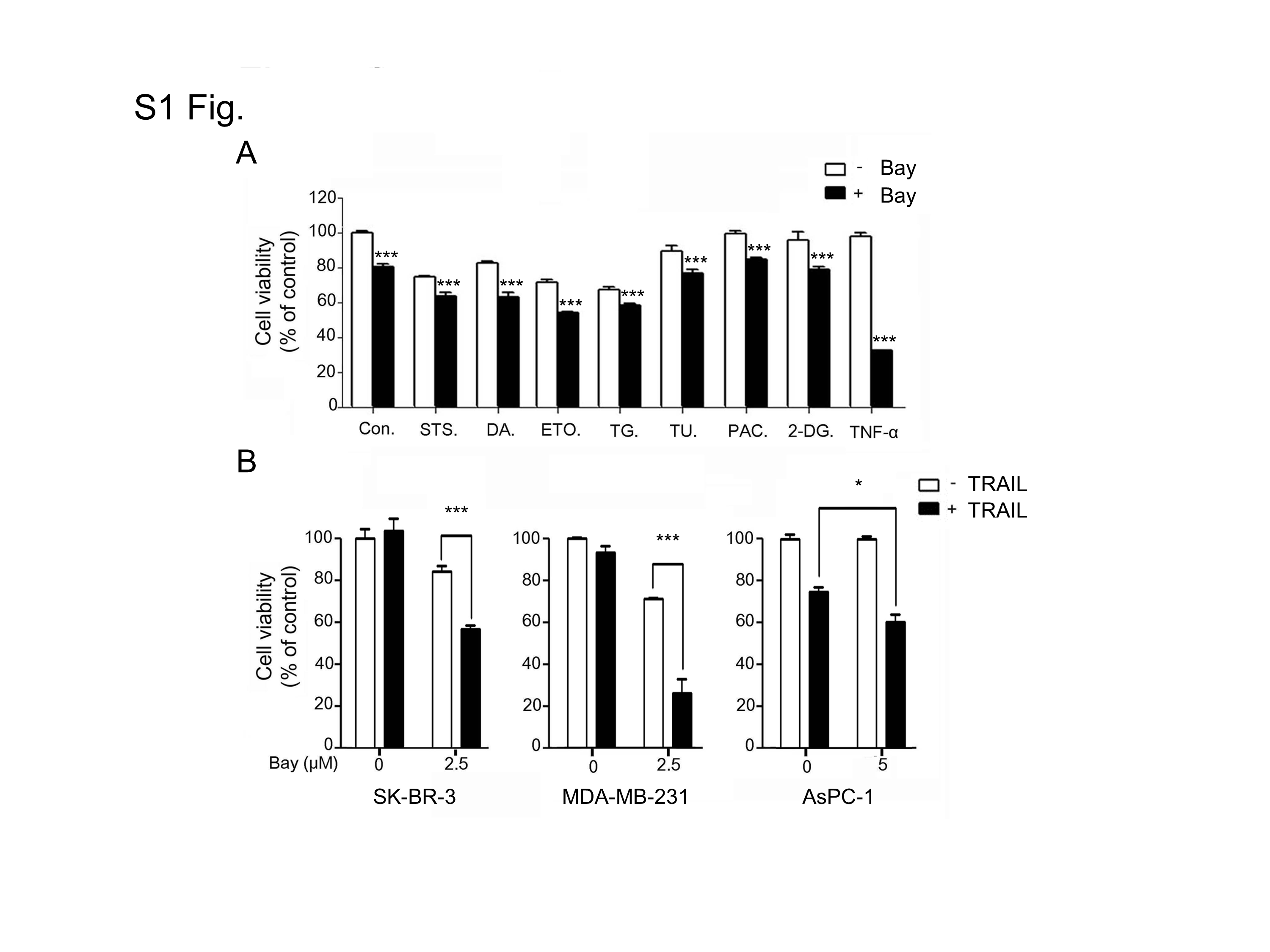

Supplement: S1 Fig — (A) Cells were exposed to Bay 61–3606 (2.5 μM) for 1 h in a 96-well plate, after which they were exposed to various cell-death-inducing agents including 125 nM staurosporine (STS), 5 μM daunorubicin (DA), 800 nM etoposide (ETO), 10 μM thapsigargin (TG), 625 ng/ml tunicamycin (TUN), 2.5 ng/ml paclitaxel (PAC), 312.5 nM 2-deoxyglucose (2-DG) or 40 ng/ml tumor necrosis factor alpha (TNF-α) for 24 h. (B) The Bay 61-3606-induced sensitization was tested in SK-BR-3, MDA-MB-231, and AsPC-1 cells which are resistant to TRAIL (50 ng/ml). Asterisks indicate significant differences compared with the control (* P <0.05, ** P <0.01, and *** P <0.001). (TIF) [file pone.0146073.s001.tif]

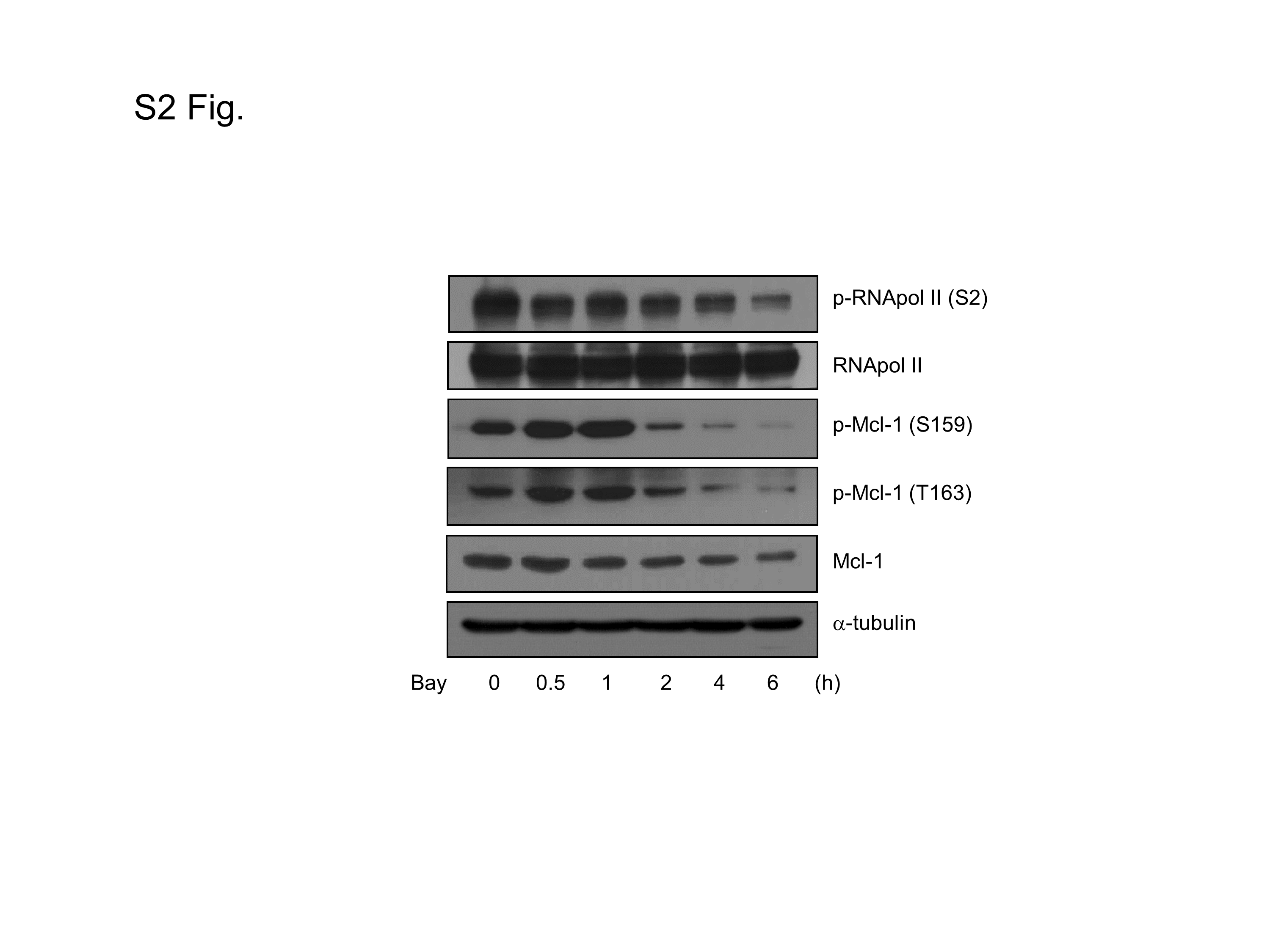

Supplement: S2 Fig — MCF-7 cells were exposed to Bay 61–3606 (2.5 μM) for increasing times, and cell extracts were analyzed by Western blotting using the indicated antibodies. (TIF) [file pone.0146073.s002.tif]

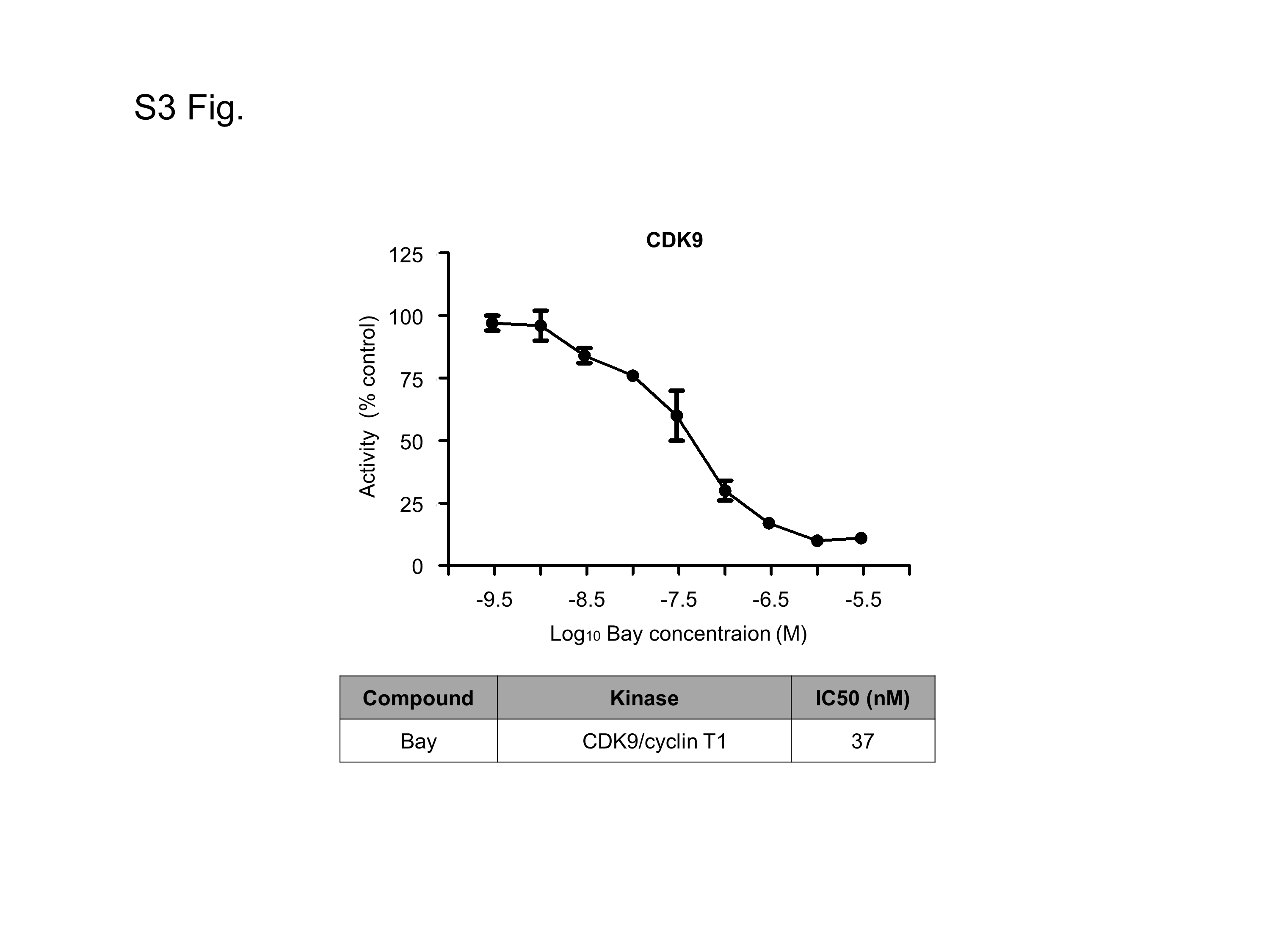

Supplement: S3 Fig — Kinase activity and in vitro IC50 were determined by Merck Millipore’s Kinase Profiling Service. Protein kinase was tested in a radiometric assay format, and the raw data was measured by scintillation counting. (TIF) [file pone.0146073.s003.tif]

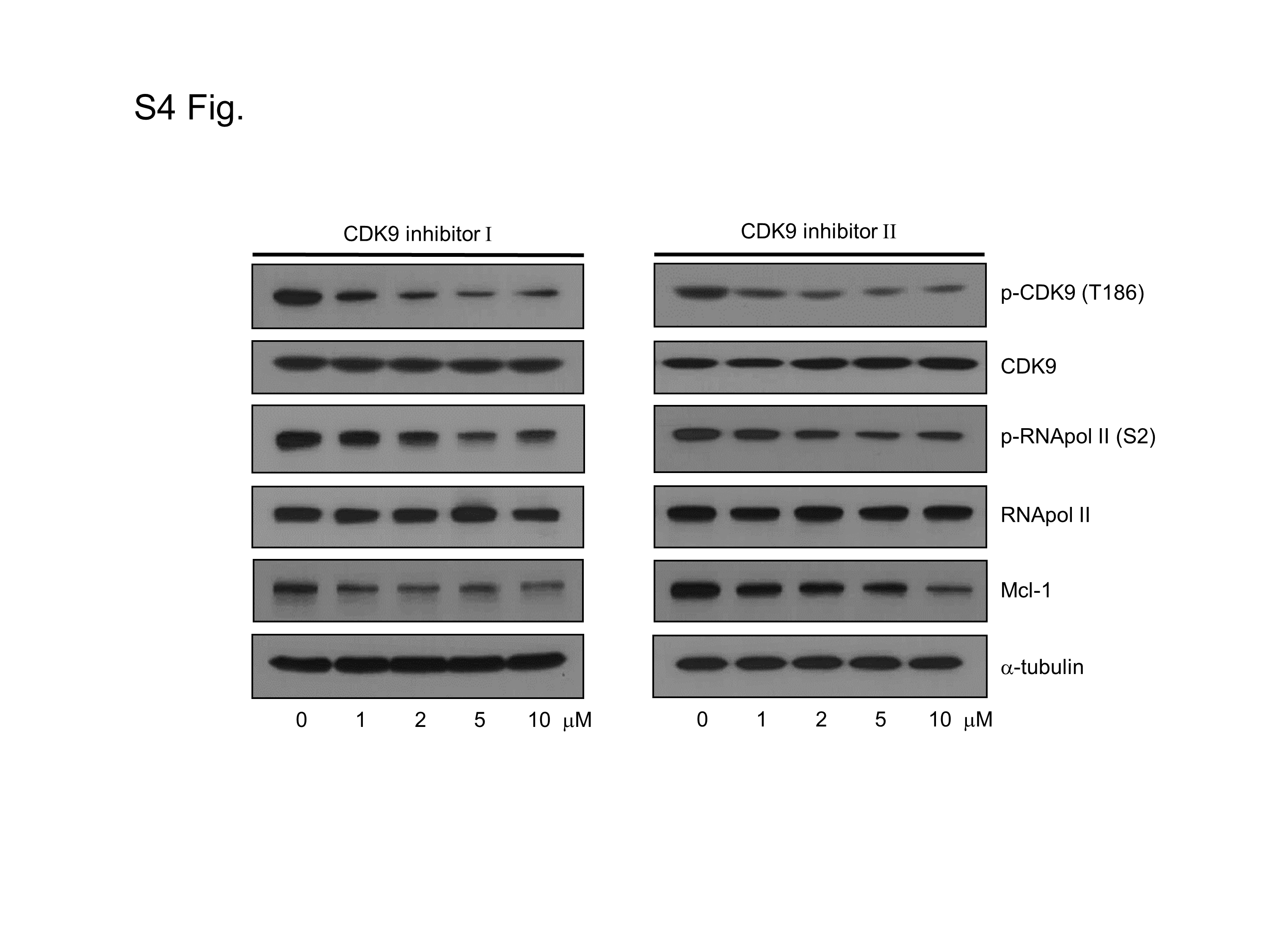

Supplement: S4 Fig — MCF-7 cells were treated with the indicated concentrations of two CDK9 inhibitors (I and II) for 1 h, after which cell extracts were analyzed by Western blotting using the indicated antibodies. (TIF) [file pone.0146073.s004.tif]

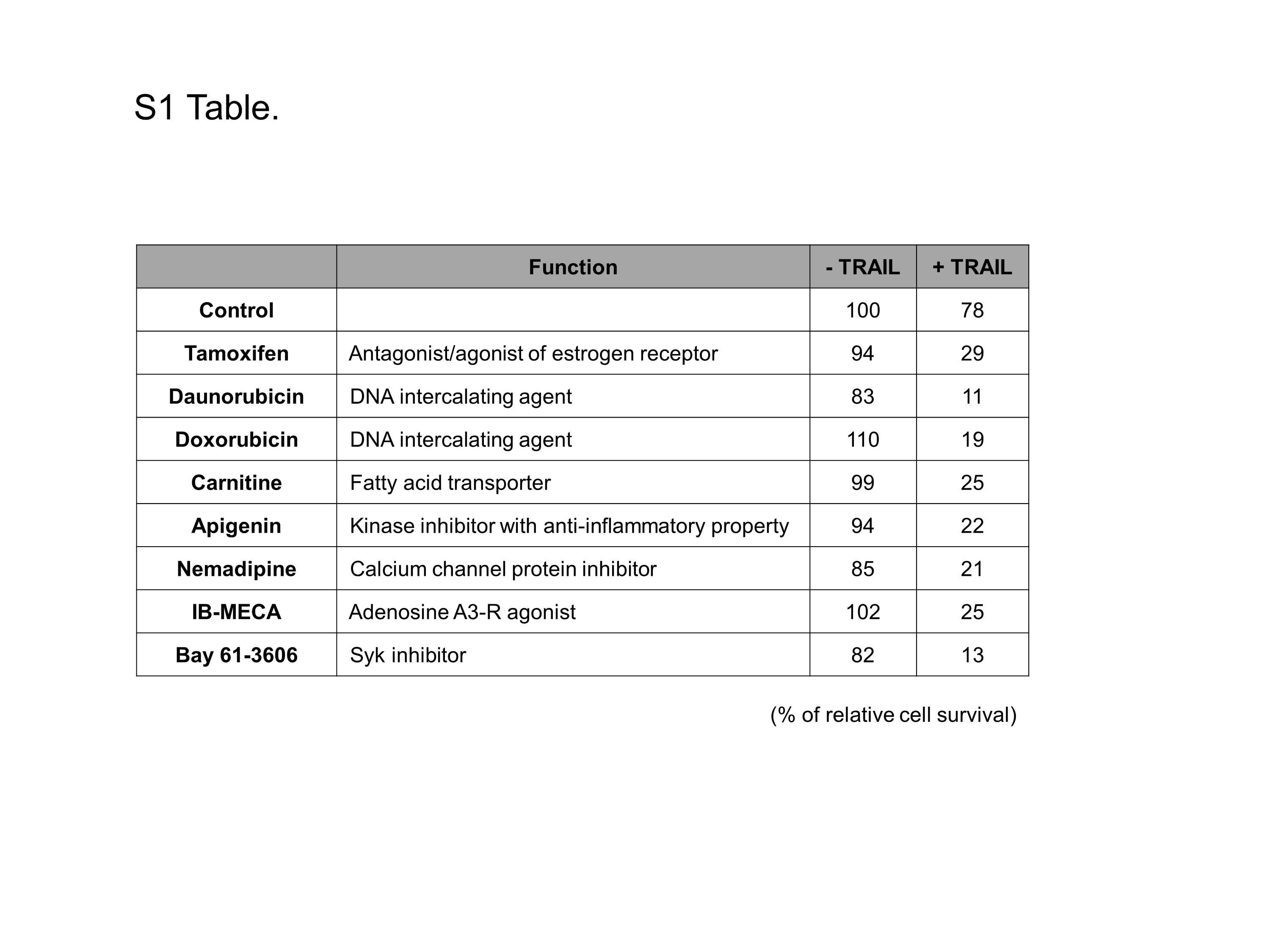

Supplement: S1 Table — After pre-incubation of the test compounds (5 μM), cells were exposed to TRAIL (50 ng/ml) for 24 h. Relative cell survival was determined by assaying the ATP levels. (TIF) [file pone.0146073.s005.tif]
